# Heterogeneous light environments induce plasticity in leaf morphology and photosynthesis of two sympodial bamboo species

**DOI:** 10.3389/fpls.2026.1803095

**Published:** 2026-04-22

**Authors:** Xin Deng, Xia Jiang, Hongming Zhong, Xiangjie Zeng, Xuanyu Tao, Yongquan Ren

**Affiliations:** 1 College of Eco-Environmental Engineering, Guizhou Minzu University, Guiyang, China; 2 Guizhou Liping Rocky Desertification Ecosystem Observation and Research Station, Guizhou Academy of Forestry, Guiyang, China; 3 Chishui National Forest Germplasm Bank of Bamboo, Chishui Forestry Bureau, Chishui, China

**Keywords:** Adaptative strategy, leaf mass per area, light heterogeneity, morphological plasticity, resource allocation, sympodial bamboo

## Abstract

**Introduction:**

Light heterogeneity is common in forest ecosystems. It arises from both horizontal (orientation) and vertical (canopy layer) gradients, driving adaptive strategies in plant morphology and physiology.

**Methods:**

The responses of leaf morphological and physiological traits in *Dendrocalamus farinosus* and *Bambusa rigida* were studied to clarify the adaptative mechanism of these two sympodial bamboo species to heterogeneous light environments.

**Results and discussion:**

These two bamboo species showed a distinctly higher leaf mass per area (LMA) under high-light environments, with the highest LMA in upper canopy layer of south orientation and the lowest LMA in lower canopy layer of north orientation. Similarly, the maximum net photosynthetic rate (P_nmax_) of both bamboo species was significantly higher in high-light environments (south orientation and upper layer) than in low-light environments (north orientation and lower layer). However, the two sympodial bamboo species demonstrated distinct acclimation strategies in leaf morphological and photosynthetic traits: *D. farinosus* achieved broad acclimation through the stability of its functional traits, whereas *B. rigida* attained precise acclimation via morphological and physiological plasticity. This study reveals a consistent increase in leaf investment under high light conditions in two sympodial bamboos, alongside species-specific variations in the extent of their morphological and physiological plasticity. These findings provide a theoretical basis for the sustainable management of bamboo forest ecosystems.

## 1 Introduction

Light serves as the sole energy source for plant photosynthesis and is also a key environmental factor that shapes plant morphogenesis (Wang et al., 2021). In natural ecosystems, particularly in structurally complex forests, intricate light gradients arise from both the vertical layering of the canopy and horizontal variations due to factors such as slope aspect, forest edge effects, and shading from neighboring vegetation (Matsuo et al., 2021; Graham et al., 2021; Keuskamp et al., 2010). To adapt to such light gradients, plants can adjust their resource allocation strategies to optimize light capture and utilization efficiency (Chen et al., 2022; Granata et al., 2020).

Leaf is the primary organ for plant photosynthesis, and the plasticity of leaf traits is highly sensitive to changes in the light environment (Li et al., 2023; Odé et al., 2025). Under low-light conditions, plants often increase leaf area to enhance light capture potential, thereby increasing the efficiency of light use (He et al., 2025; Yu et al., 2023). Conversely, in high-light environments, a smaller leaf area helps reduce the risk of photodamage caused by excessive radiation (Lanoue et al., 2022; Ren et al., 2021). The decrease in leaf mass per area (LMA) under shade represents a resource−allocation strategy to reduce material investment per unit area, optimizing the balance between carbon assimilation and construction costs when light is limited (Mizell et al., 2025). Chlorophyll content, a key physiological parameter regulating light energy absorption and conversion, tends to increase in low-light conditions, enabling plants to enhance light capture and utilization efficiency (Khoshbakht et al., 2018; Zhang et al., 2017). Moreover, the photosynthetic light−response curve provides a direct quantitative measure of leaf photosynthetic capacity across varying light intensities, illustrating the trade−off between photosynthetic production and respiratory consumption in plants (de Santana et al., 2025). The coordinated variation of these leaf traits across light gradients represents a critical adaptive strategy for plants to cope with heterogeneous light environments.

As a significant global forest resource, bamboo holds substantial ecological and economic value. Sympodial bamboos grow in characteristic clonal clusters, composed of numerous culms of different ages and sizes that collectively form a vertically stratified canopy structure (Xu et al., 2025). Within each cluster, the upper canopy is exposed to full sunlight while the lower layer remain shaded, establishing a distinct vertical light gradient. Furthermore, bamboo clusters at the forest edge receive higher solar radiation than interior ones, while radiation levels among these edge clusters further vary according to their orientation. In the northern hemisphere, individuals at the southern edge generally experience more intense direct sunlight than those at the northern side (Zhang et al., 2024). Due to its unique biological traits, sympodial bamboo serves as an excellent experimental system for investigating how light heterogeneity influences plant resource allocation.

To date, studies of the plasticity in leaf traits under varying light conditions have primarily focused on broad-leaved trees, crops, or monopodial bamboos (Catoni et al., 2015; Yang et al., 2014; Zhang et al., 2022). In addition, species exhibit varied responses to changes in light environment. For example, while the average leaf area of some tree species increases from the high−light upper canopy to the shaded lower canopy, other species exhibit a decline from the moderately lit middle canopy to the low−light understory (Catoni et al., 2015). Although studies have examined the effects of vertical light gradients on leaf traits in certain bamboo species (Cao et al., 2013; Gao et al., 2023; Yang et al., 2014), an integrated analysis incorporating both vertical and horizontal light gradients in sympodial bamboos remains lacking. This study investigated two typical sympodial bamboo species, measuring key morphological and photosynthetic traits at both northern and southern orientations and across upper and lower canopy layers. The objectives were to (1) analyze how leaf traits vary under different light conditions, and (2) elucidate the role of light heterogeneity in driving resource allocation strategies in sympodial bamboos. The results will deepen our understanding of the adaptive mechanisms of sympodial bamboos to heterogeneous light environments and provide a theoretical foundation for the sustainable management of bamboo forests.

## 2 Materials and methods

### 2.1 Study site

This study was conducted in Chishui national forest germplasm bank of bamboo, located in the northwestern Guizhou province, SW China (105°69′17″E, 28°54′54″N, 310 m a.s.l.). The site experiences a mid-subtropical humid monsoon climate, with a mean annual temperature of approximately 18 °C. Annual precipitation is around 1200 mm, predominantly occurring between April and October. Soils in the experimental area are predominantly purple and yellow, characterized by a loose texture and weak acidity. These properties provide favorable conditions for bamboo growth.

### 2.2 Experimental materials

**Figure 1:**
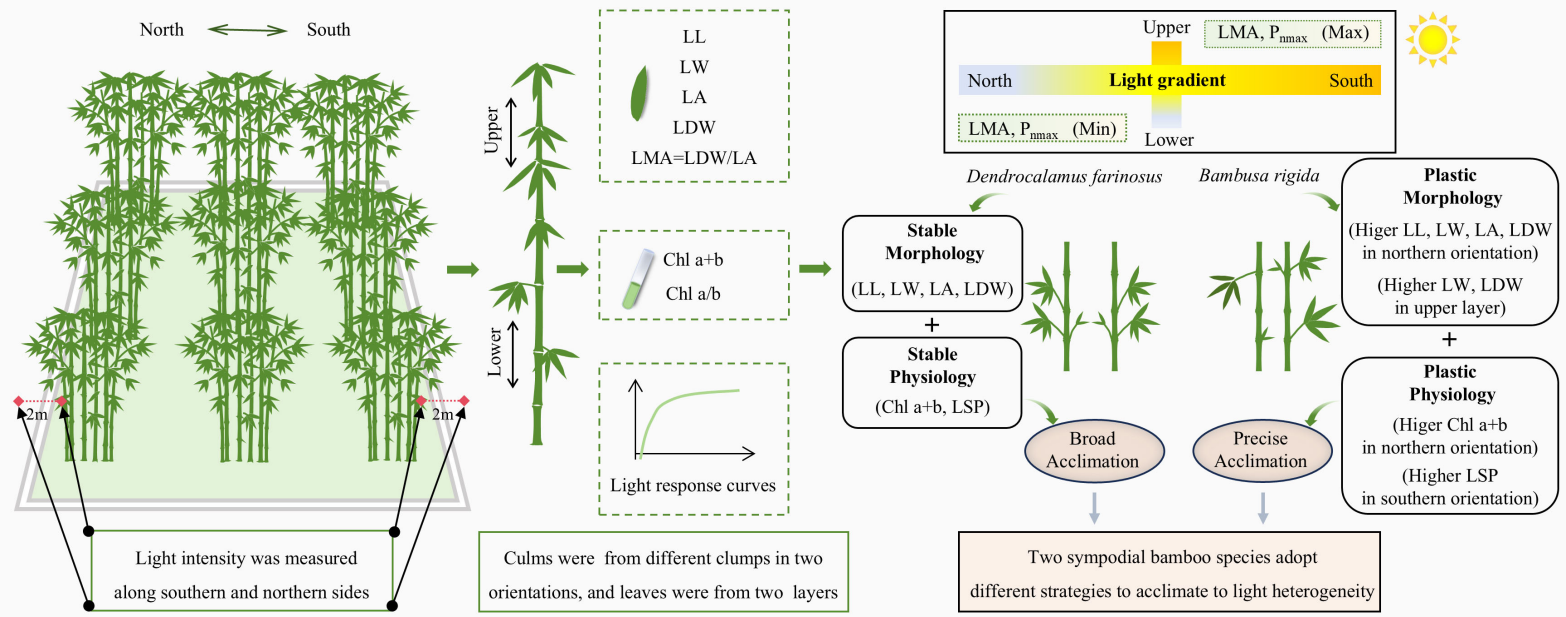
Summary figure illustrating the sampling, measurements, and key findings of this study. For each bamboo species, clumps were planted in a square nursery, with its four sides precisely aligned with the four directions (east, north, west, and south). Light intensity was measured along the northern and southern edges. To assess morphological and physiological traits, bamboo culms were selected from different clumps, considering two orientations and two canopy layers. Morphological traits of leaf length (LL), leaf width (LW), leaf area (LA), leaf dry weight (LDW) and leaf mass per area (LMA) were measured. Total chlorophyll content (Chl a+b) was measured and the ratio of chlorophyll a to chlorophyll b (Chl a/b) was calculated, and the photosynthetic light response curves of leaves were also fitted. LSP: light saturation point.

*Dendrocalamus farinosus* and *Bambusa rigida*, two economically important bamboo species in southern China, were selected as the research subjects. Both species were planted in square nurseries of 20 m × 20 m, with the four sides of the nurseries located exactly in the four directions of east, north, west, and south. Healthy culms were selected from different clumps to measure morphological and physiological traits (**Figure 1**). Based on preliminary observations, and to ensure the measurement of fully mature and stable leaf traits, different physiological maturity ages were adopted for the two species: two-year-old culms were sampled for *D. farinosus*, whereas one-year-old culms were used for *B. rigida*.

### 2.3 Light intensity

Light intensity was measured with a DL333205 digital lux meter (Deli Group Co., Ltd., Ningbo, China) at 30−minute intervals between 9:30 AM and 12:00 PM. Measurements were conducted on two consecutive sunny days, with one day dedicated to each species. Sampling positions were located along the southern and northern sides of the bamboo forest. During each measurement, the sensor of the illuminance meter was held horizontally and kept unobstructed. It was first placed directly above a lower-canopy leaf to record the actual light intensity incident on the leaf. To account for shading from upper-canopy foliage, the sensor was then moved horizontally outward by 2 meters while maintaining the same height. On both southern and northern orientations, three independent replicates were established at each sampling position, spaced 2 m apart.

### 2.4 Leaf morphological traits

For each species, five culms from different clumps were randomly selected on both the southern and northern orientations of the forest to serve as replicates. From each culm, branches were then sampled from both the upper and lower canopy layers. From these branches, fully expanded leaves (the second to third from the apex) were collected. The number of leaves collected was determined according to the single leaf area of the two species: eight leaves for *D. farinosus* and ten leaves for *B. rigida* from each layer of each culm. Prior to measurement, the leaf petioles were removed. Each leaf was then placed flat on a white paper alongside a scale ruler and photographed using a smartphone held parallel to the paper surface. The images were analyzed with ImageJ software (National Institutes of Health, Bethesda, MD, USA) to determine leaf length (LL, cm), leaf width (LW, cm), and leaf area (LA, cm²). Following imaging, all leaves were placed in paper envelopes and oven-dried at 65 °C until constant weight was achieved to obtain leaf dry weight (LDW, g). For each culm, leaf morphological trait measurements were averaged per canopy layer to obtain a single representative value. Based on these mean values, the leaf mass per area (LMA, g·m^-^²) was subsequently calculated as LMA = LDW/LA.

### 2.5 Leaf physiological traits

Following the same sampling procedure used for morphological measurements, leaves were collected from the same five replicate culms for chlorophyll content analysis. These leaves were immediately wrapped in aluminum foil and placed in a foam box containing dry ice. The total chlorophyll (Chl a+b) content was subsequently determined using the acetone−ethanol mixed extraction method as described by Wellburn (Liao et al., 2023). The ratio of chlorophyll a to chlorophyll b (Chl a/b) was calculated as Chl a/b = Chl a/Chl b. For each replicate culm, chlorophyll content for each canopy layer was determined on pooled leaf samples. The photosynthetic light response curves of leaves were measured using a portable photosynthesis system (Li-6400XT, Li-Cor, Inc., Lincoln, NE, USA). For each orientation and layer, three leaves were sampled from three different culms as replicates. Prior to formal measurements, leaves were pre-conditioned under an irradiance of 1500 μmol·m^-^²·s^-^¹ to fully activate the photosynthetic apparatus. Photosynthetically active radiation (PAR) was then set in a descending sequence of 12 levels: 2000, 1500, 1200, 1000, 800, 600, 400, 200, 100, 50, 20, and 0 μmol·m^-^²·s^-^¹. The minimum and maximum stabilization times at each light level were 120 s and 180 s, respectively.

### 2.6 Data analysis

As all leaf traits were continuous and positive, we fitted a generalized linear model (GLM) with a Gamma distribution and a log-link function to assess the effects of orientation, canopy layer. Diagnostic checks, including residual plots and dispersion tests, confirmed that the residuals were randomly distributed and conformed to the assumptions of the Gamma GLM. Photosynthetic light response curves were fitted using a non-rectangular hyperbola model (Zhu et al., 2020). The robustness of the fitting parameters was confirmed by high coefficients of determination, with R² values ranging up to 0.998. Data were analyzed using SPSS version 27 (IBM Corp., Armonk, NY, USA). All figures were prepared with Origin 2024 software (Origin Lab, Northampton, MA, USA).

## 3 Results

### 3.1 Light intensity

**Figure 2:**
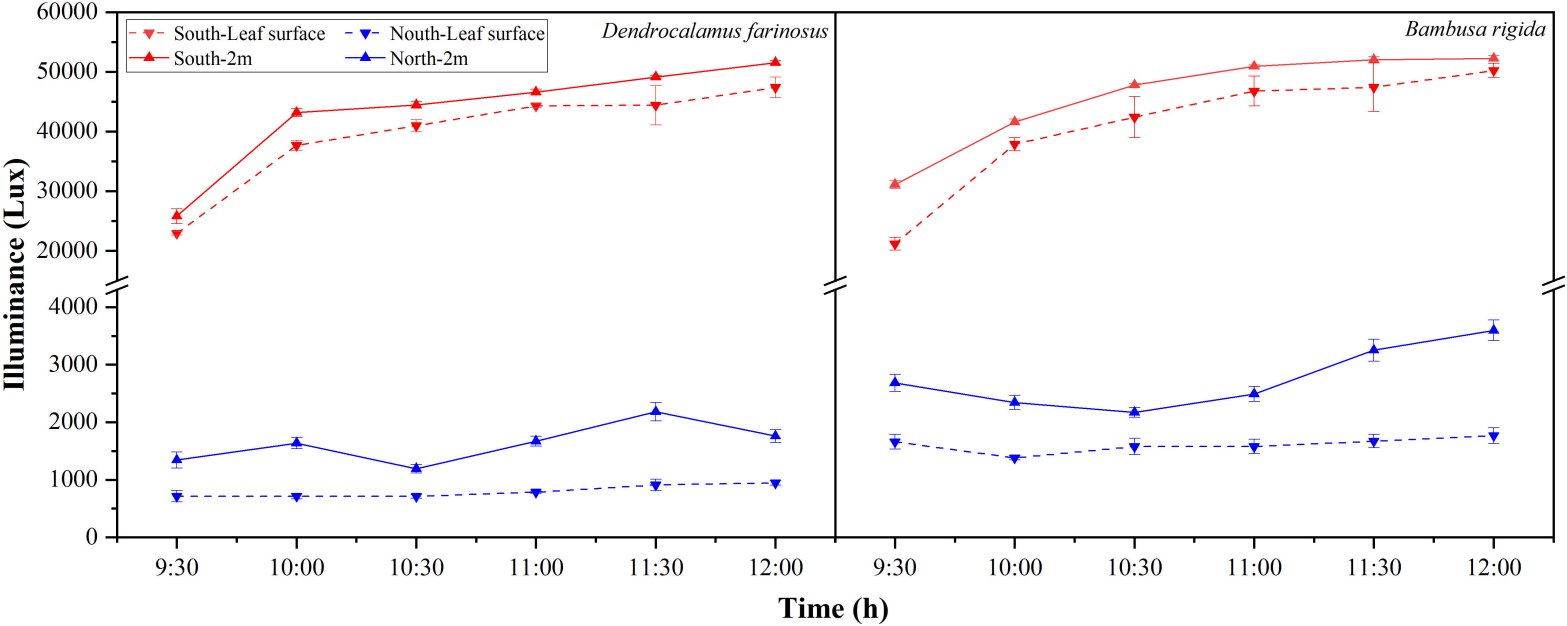
Light intensity distribution within *Dendrocalamus farinosus* and *Bambusa rigida* forests from 9:30 AM to 12:00 PM. Measurements were taken at four distinct positions: directly above a lower-canopy leaf at the south-facing edge (South-leaf surface), and 2 m horizontally outward from the south-facing leaf (South-2 m), directly above a lower-canopy leaf at the north-facing edge (North-leaf surface), and 2 m horizontally outward from the same leaf (North-2 m). Data points represent means, and error bars represent the standard error of the mean (*n* = 3).

For both species, light intensity measured from 9:30 AM to 12:00 PM differed spatially and temporally between the two orientations (**Figure 2**). In the south, light intensity rose progressively over time, while in the north it remained stable at a low level. Consequently, light intensity was significantly higher in the south than in the north (*p* < 0.05). Moreover, measurements taken 2 m outward from the edge exceeded those directly above lower-layer leaves in both orientations.

### 3.2 Morphological traits

**Figure 3:**
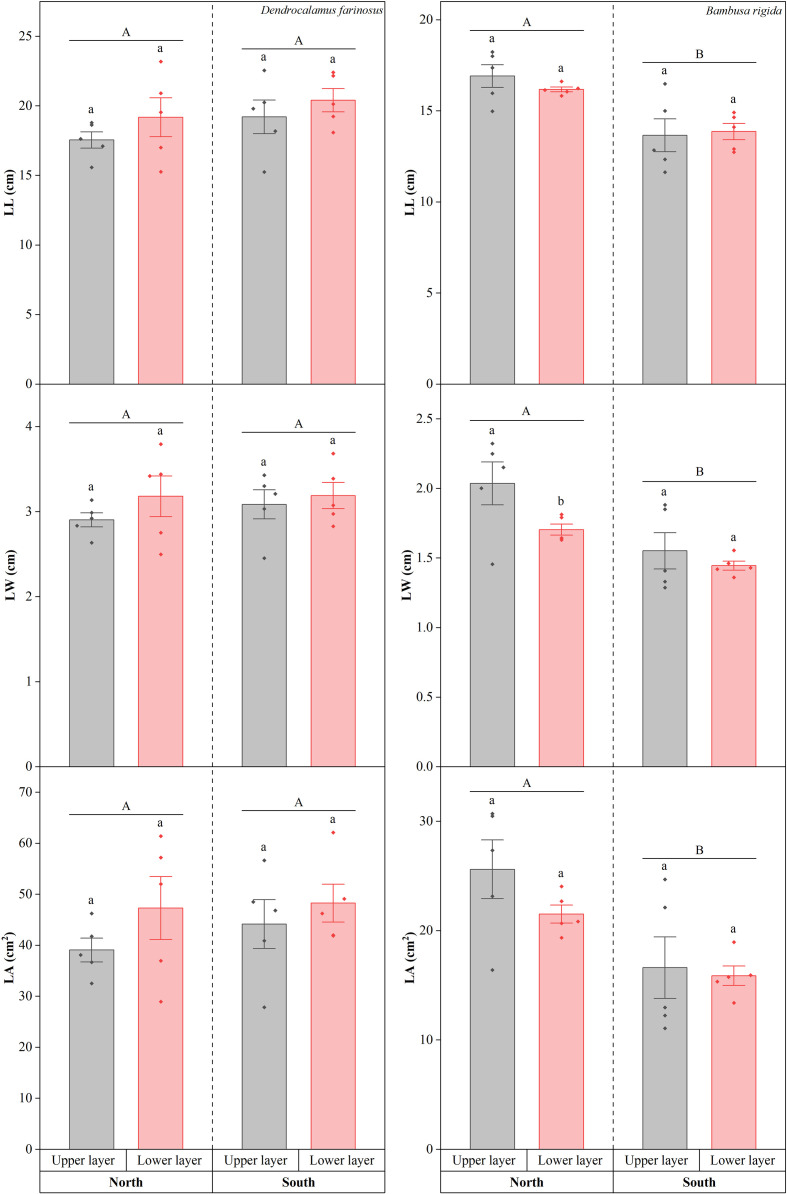
Leaf length (LL), leaf width (LW) and Leaf area (LA) of *Dendrocalamus farinosus* and *Bambusa rigida* across orientations and canopy layers. Error bars represent the standard error of the mean (*n* = 5). Shared or different uppercase letters indicate no significant or significant differences between orientations (*p* = 0.05), while shared or different lowercase letters denote no significant or significant differences between canopy layers within the same orientation (*p* = 0.05).

For *D. farinosus*, both LL and LW showed no significant differences between southern and northern orientations (LL: Wald *χ*^2^ = 2.328, *df* = 1, *p* = 0.127; LW: Wald *χ*^2^ = 0.388, *df* = 1, *p* = 0.534) or between upper and lower canopy layers (LL: Wald *χ*^2^ = 2.244, *df* = 1, *p* = 0.134; LW: Wald *χ*^2^ = 1.551, *df* = 1, *p* = 0.213; **Figure 3**). For *B. rigida*, significant differences were detected in both LL and LW between the two orientations (LL: Wald *χ*^2^ = 27.283, *df* = 1, *p* < 0.001; LW: Wald *χ*^2^ = 15.910, *df* = 1, *p* < 0.001). However, only LW was significantly affected by canopy layer (Wald *χ*^2^ = 5.534, *df* = 1, *p* = 0.019), while LL remains unaffected (Wald *χ*^2^ = 0.250, *df* = 1, *p* = 0.617).

The responses of LA in both species to orientations and canopy layers followed the patterns similar to those observed for LL (**Figure 3**). LA of *D. farinosus* was not significantly influenced by orientation (Wald *χ*^2^ = 0.568, *df* = 1, *p* = 0.451) or canopy layer (Wald *χ*^2^ = 2.374, *df* = 1, *p* = 0.123). In both the northern and southern orientations, no significant difference was observed between the upper and lower canopy layers (all *p* > 0.05). In *B. rigida*, LA did not differ significantly between canopy layers (Wald *χ*^2^ = 1.765, *df* = 1, *p* = 0.184). However, LA was significantly higher in the north compared to the south (Wald *χ*^2^ = 16.139, *df* = 1, *p* < 0.001).

**Figure 4:**
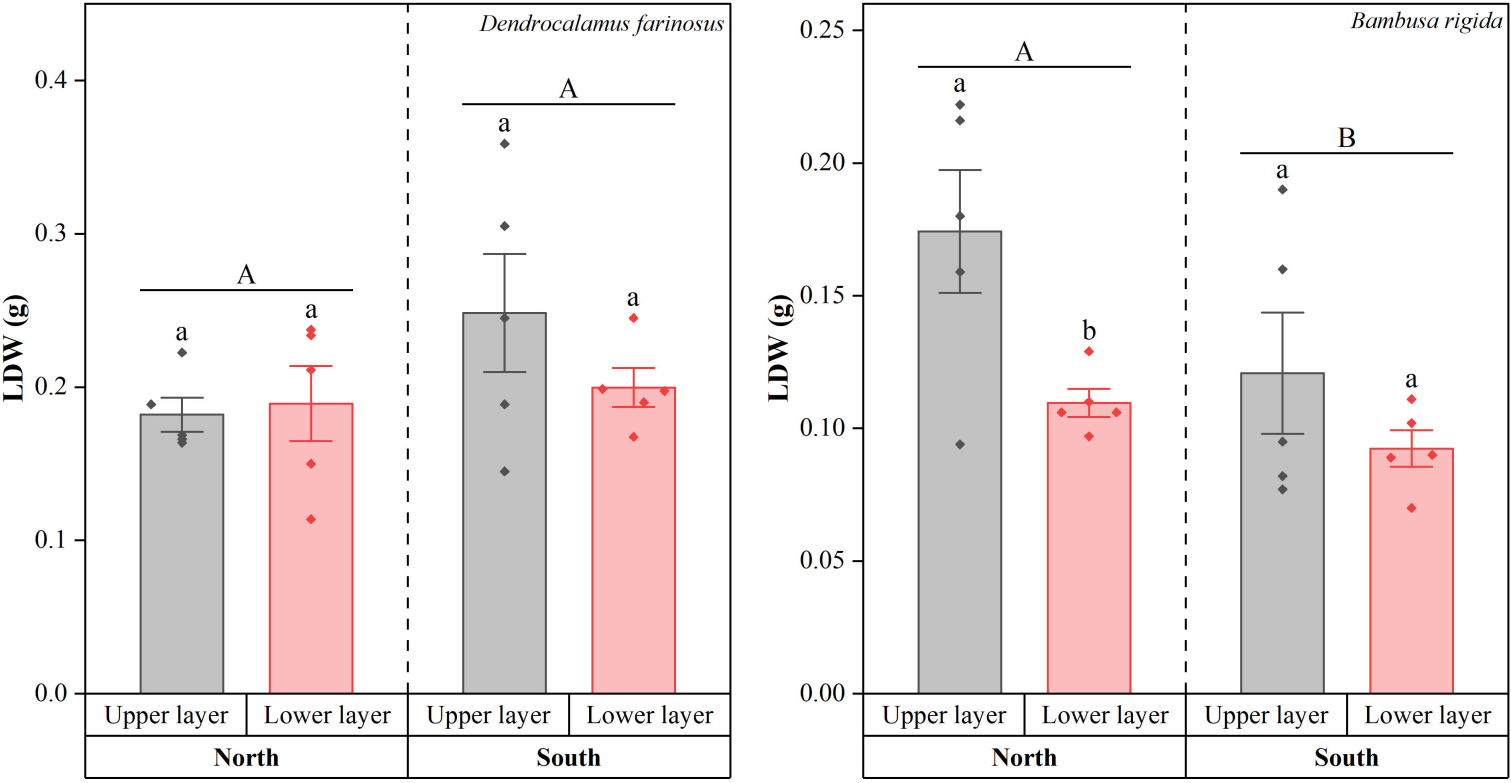
Leaf dry weight (LDW) of *Dendrocalamus farinosus* and *Bambusa rigida* across orientations and canopy layers. Error bars represent the standard error of the mean (*n* = 5). Shared and different uppercase letters denote no significant or significant differences between orientations (*p* = 0.05), while shared or different lowercase letters indicate no significant or significant differences between canopy layers within each orientation (*p* = 0.05).

Consistent with the results of LA, LDW of *D. farinosus* was significantly influenced by neither orientation (Wald *χ*^2^ = 3.133, *df* = 1, *p* = 0.077) nor canopy layer (Wald *χ*^2^ = 0.910, *df* = 1, *p* = 0.340; **Figure 4**). In both the northern and southern orientations, LDW did not differ significantly between upper and lower canopy layers (all *p* > 0.05). In contrast, the response of LDW in *B. rigida* differed from that of LA across orientations and canopy layers. Both orientation (Wald *χ*^2^ = 5.499, *df* = 1, *p* = 0.019) and canopy layer (Wald *χ*^2^ = 9.541, *df* = 1, *p* = 0.002) significantly affected LDW in *B. rigida*. Specifically, its LDW was significantly higher in the upper layer than in the lower layer in the north (*p* < 0.05), while no significant difference was observed between the layers in the south (*p* > 0.05).

**Figure 5:**
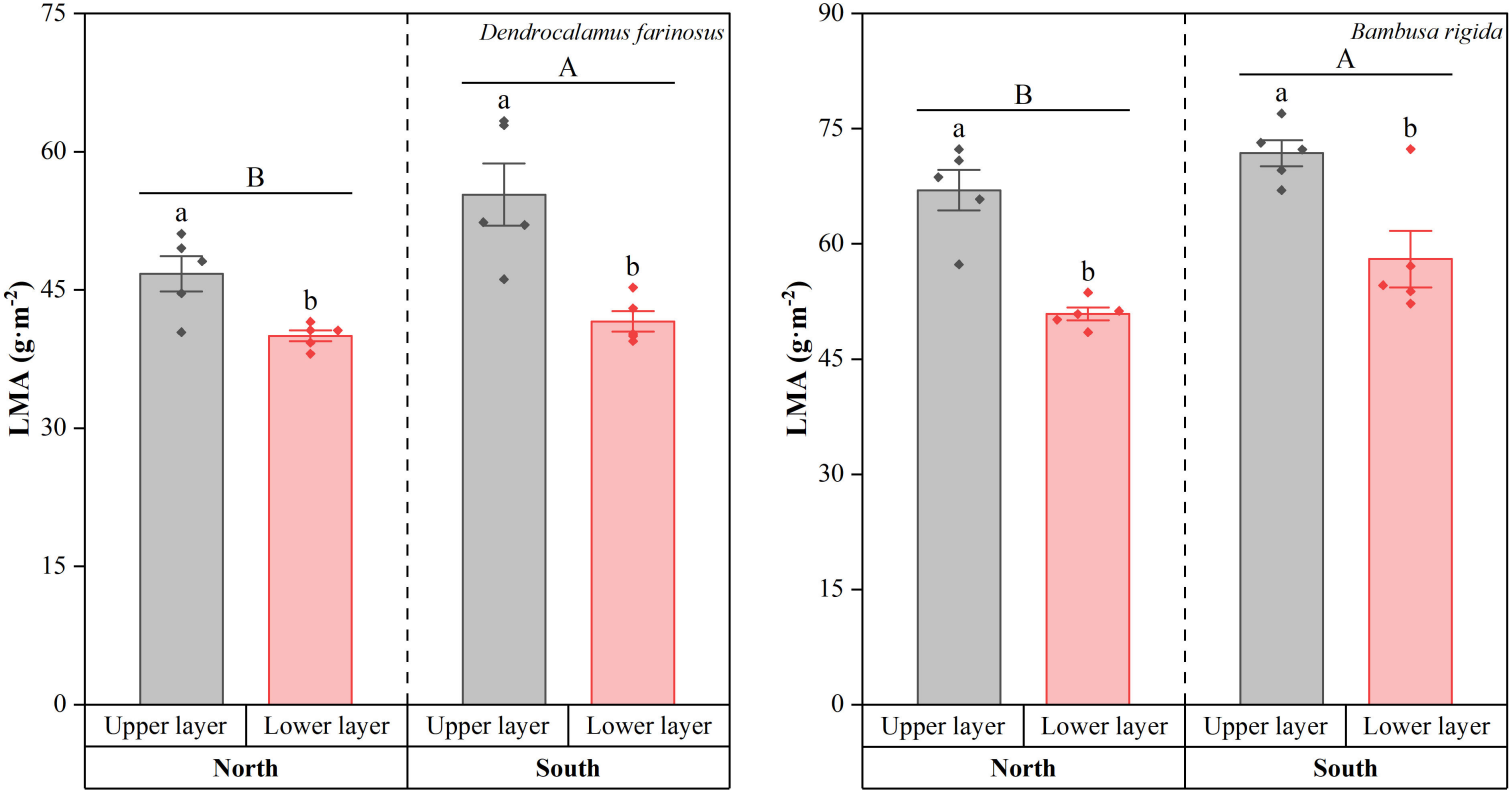
Leaf mass per area (LMA) of *Dendrocalamus farinosus* and *Bambusa rigida* across canopy layers and orientations. Error bars represent the standard error of the mean (*n* = 5). Different uppercase letters indicate significant differences between orientations (*p* < 0.05), and different lowercase letters denote significant differences among canopy layers within each orientation (*p* < 0.05).

In *D. farinosus*, LMA differed significantly with orientation (Wald *χ*^2^ = 7.815, *df* = 1, *p* = 0.005) and canopy layer (Wald *χ*^2^ = 31.851, *df* = 1, *p* < 0.001; **Figure 5**). Similarly, in *B. rigida*, orientation (Wald *χ*^2^ = 7.426, *df* = 1, *p* = 0.006) and canopy layer (Wald *χ*^2^ = 46.563, *df* = 1, *p* < 0.001) also significantly influenced LMA. Overall, LMA was significantly higher on the southern orientation than the northern orientation, and higher in the upper canopy layer than in the lower layer (all *p* < 0.05). Consequently, the highest LMA occurred in the upper canopy of the southern orientation, while the lowest was recorded in the lower canopy of the northern orientation.

### 3.3 Physiological traits

**Figure 6:**
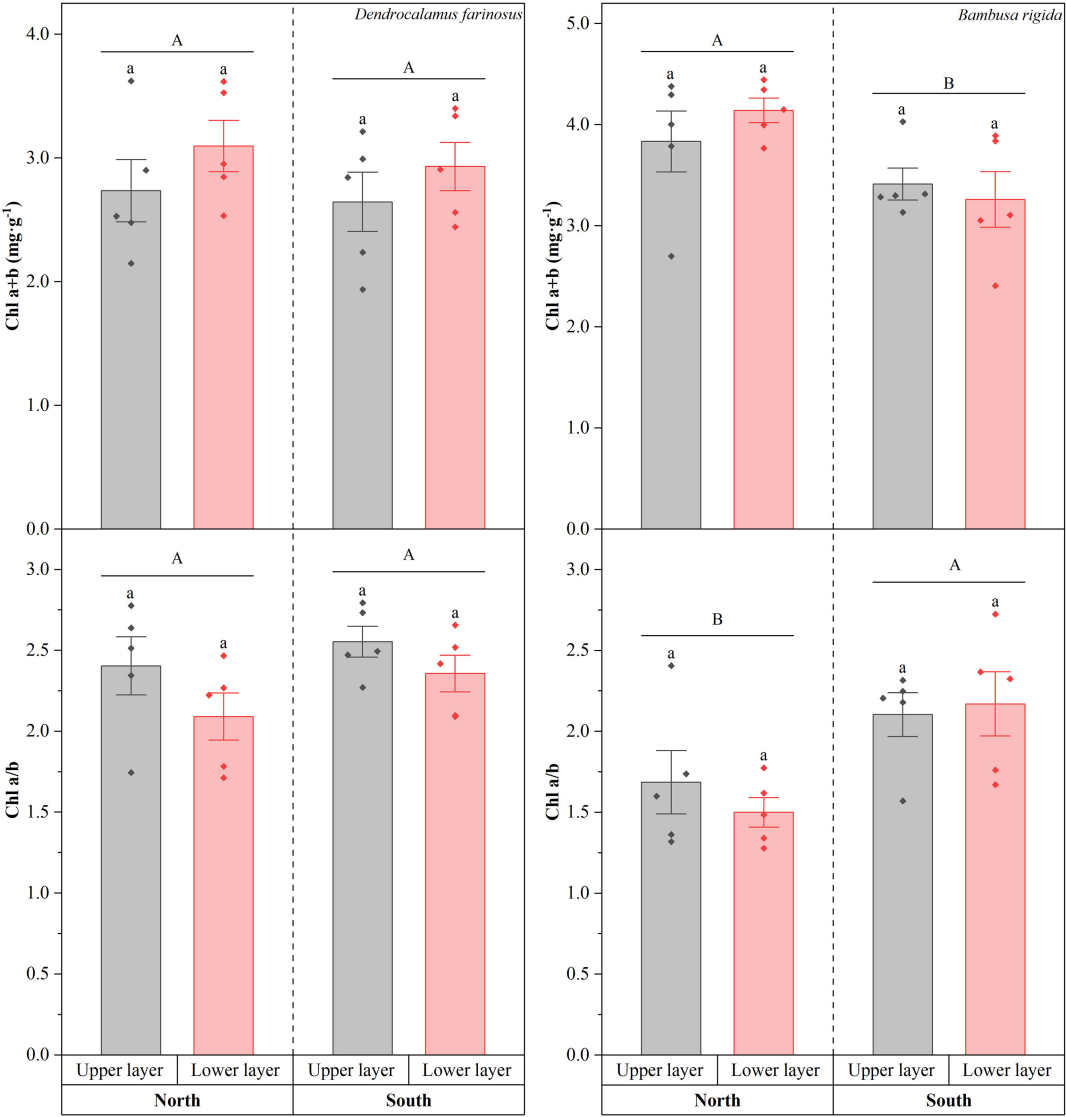
Total chlorophyll content (Chl a+b), chlorophyll a/b ratio (Chl a/b) of *Dendrocalamus farinosus* and *Bambusa rigida* in different orientations and canopy layers. Error bars represent the standard error of the mean (*n* = 5). Shared or different uppercase letters indicate no significant or significant differences between orientations (*p* = 0.05), and same lowercase letters indicate no significant differences between canopy layers in each orientation (*p* > 0.05).

These two species exhibited distinct response patterns to light environment in terms of chlorophyll content and its components (**Figure 6**). In *D. farinosus*, there was no significant difference in Chl a+b content between two orientations (Wald *χ*^2^ = 0.408, *df* = 1, *p* = 0.523) or canopy layers (Wald *χ*^2^ = 2.578, *df* = 1, *p* = 0.108). Differently, in *B*. *rigida*, the Chl a+b content was significant higher in the north compared to the south (Wald *χ*^2^ = 10.226, *df* = 1, *p* = 0.001), while that was not significantly affected by canopy layer (Wald *χ*^2^ = 0.144, *df* = 1, *p* = 0.704). The chlorophyll a/b ratio of both bamboo species also responded differently to canopy layers and orientations (**Figure 6**). In *D. farinosus*, the Chl a/b ratio was significantly influenced by canopy layer (Wald *χ*^2^ = 4.299, *df* = 1, *p* = 0.038), with higher values recorded in the upper layer, but not by orientation (Wald *χ*^2^ = 2.848, *df* = 1, *p* = 0.091). In *B. rigida*, the Chl a/b ratio was significantly influenced by orientation (Wald *χ*^2^ = 14.209, *df* = 1, *p* < 0.01), with higher values recorded on the southern orientation, but not by canopy layer (Wald *χ*^2^ = 0.172, *df* = 1, *p* = 0.678).

**Figure 7:**
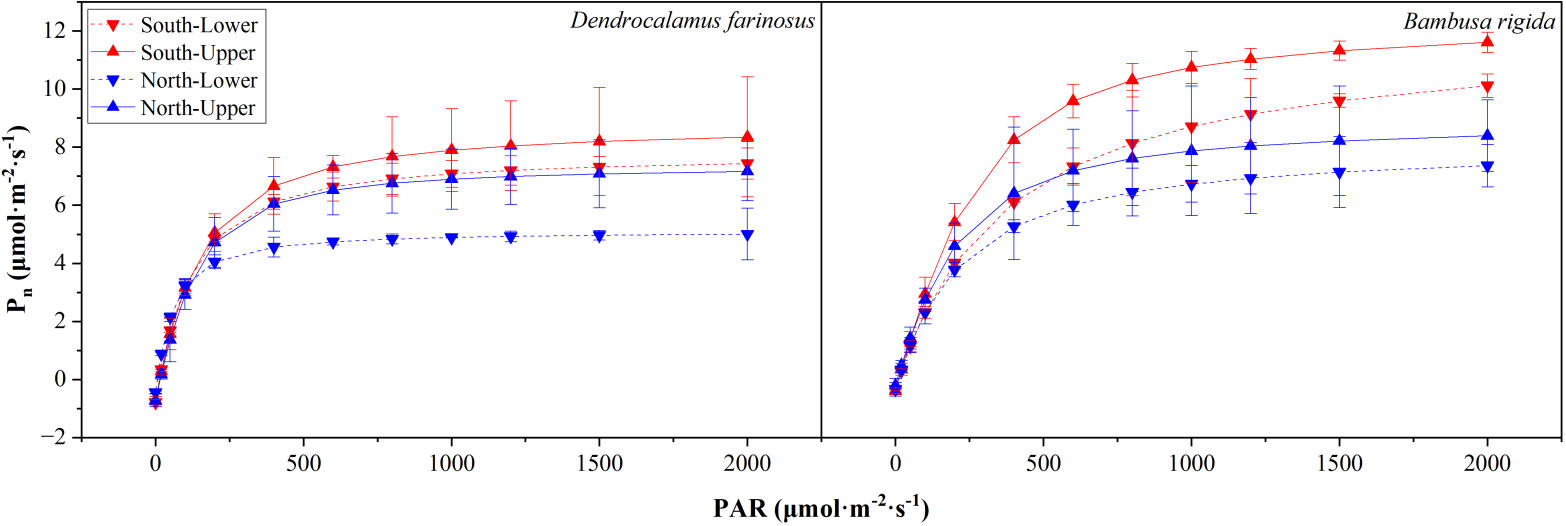
Photosynthetic light−response curves of *Dendrocalamus farinosus* and *Bambusa rigida* across different orientations and canopy layers. P_n_, net photosynthetic rate; PAR, photosynthetically active radiation. Curves were fitted at four distinct positions: lower canopy in southern orientation (South-Lower), upper canopy in southern orientation (South-Upper), lower canopy in northern orientation (North-Lower), and upper canopy in northern orientation (North-Upper). Error bars represent the standard error of the mean (*n* = 3).

For both *D. farinosus* and *B. rigida*, the net photosynthetic rate (P_n_) of leaves from different positions increased with rising PAR (**Figure 7**). When PAR was below 200 μmol·m^-^²·s^-^¹, P_n_ increased approximately linearly across all four positions. Beyond this threshold, the increase in P_n_ followed a curvilinear pattern until it gradually plateaued at the light-saturation point, reaching the maximum net photosynthetic rate (P_nmax_). Under high PAR conditions (> 800 μmol·m^-2^s^-1^), leaves in the southern orientation exhibited higher P_n_ than those in the northern orientation. Within each orientation, P_n_ was consistently higher in the upper canopy layer than in the lower layer.

Although the two species exhibited broadly similar trends in photosynthetic light-response parameters, species-specific differences emerged with respect to orientation and canopy layer (**Table 1**). In *D. farinosus*, orientation significantly affected only P_nmax_ (Wald *χ*^2^ = 4.411, *df* = 1, *p* = 0.036). In contrast, for *B. rigida*, orientation had a significant effect on P_nmax_ (Wald *χ*^2^ = 11.901, *df* = 1, *p* < 0.001), light compensation point (LCP, Wald *χ*^2^ = 31.500, *df* = 1, *p* < 0.001) and light saturation point (LSP, Wald *χ*^2^ = 3.852, *df* = 1, *p* = 0.049), with higher values consistently observed on the southern orientation. Regarding canopy layer, significant effects were detected in *D. farinosus* for P_nmax_ (Wald *χ*^2^ = 4.354, *df* = 1, *p* = 0.037), LCP (Wald *χ*^2^ = 12.500, *df* = 1, *p* < 0.001) and dark respiratory rate (R_d_, Wald *χ*^2^ = 3.947, *df* = 1, *p* = 0.047), all of which were higher in the upper layer. In *B. rigida*, by contrast, canopy layer significantly influenced only LCP (Wald *χ*^2^ = 8.643, *df* = 1, *p* = 0.003).

**Table 1:** Fitting parameters of photosynthetic light-response curves of *Dendrocalamus farinosus* and *Bambusa rigida* in different orientations and canopy layers.

Species	Positions		Parameters									
			AQY	Significance	P_nmax_ (μmol·m^-^²·s^-^¹)	Significance	LCP (μmol·m^-^²·s^-^¹)	Significance	LSP (μmol·m^-^²·s^-^¹)	Significance	*R_d_* (μmol·m^-^²·s^-^¹)	Significance
*Dendrocalamus farinosus*	South	Upper	0.039 ± 0.009 a	A	9.44 ± 1.61 a	A	20.00 ± 4.81 a	A	744.00 ± 238.41 a	A	0.70 ± 0.15 a	A
		Lower	0.047 ± 0.001 a		7.86 ± 0.37 a		14.67 ± 1.33 b		497.33 ± 31.18 a		0.71 ± 0.09 a	
	North	Upper	0.046 ± 0.012 a	A	7.84 ± 1.20 a	B	18.67 ± 2.67 a	A	513.33 ± 16.38 a	A	0.86 ± 0.22 a	A
		Lower	0.045 ± 0.006 a		5.86 ± 0.48 a		10.67 ± 2.67 b		397.33 ± 76.81 a		0.39 ± 0.04 b	
*Bambusa. rigida*	South	Upper	0.038 ± 0.008 a	A	12.12 ± 0.22 a	A	18.67 ± 1.33 b	A	929.33 ± 124.71 a	A	0.66 ± 0.09 a	A
		Lower	0.022 ± 0.006 a		11.50 ± 0.12 a		34.67 ± 2.67 a		1338.67 ± 312.84 a		0.75 ± 0.15 a	
	North	Upper	0.029 ± 0.008 a	A	9.12 ± 2.02 a	B	13.33 ± 3.53 a	B	817.33 ± 41.46 a	B	0.42 ± 0.23 a	A
		Lower	0.031 ± 0.008 a		7.88 ± 1.17 a		12.00 ± 4.00 a		760.00 ± 265.70 a		0.43 ± 0.23 a	

AQY, apparent quantum yield; P_nmax_, maximum net photosynthetic rate; LCP, light compensation point; LSP, light saturation point; R_d_, dark respiratory rate. Data are means and standard errors (*n* = 3). In the same species, shared and different uppercase letters denote no significant or significant differences between orientations (*p* = 0.05), while shared or different lowercase letters indicate no significant or significant differences between canopy layers within each orientation (*p* = 0.05).

## 4 Discussion

### 4.1 Spatial heterogeneity of light intensity

Although the light intensity recorded by the lux meter in this study is not a direct measure of PAR, it serves as a reliable proxy for the distribution of solar radiation and effectively captures the relative differences in light intensity between our sampling positions. Light distribution in both bamboo forests demonstrated significant spatial heterogeneity, shaped by both orientation and canopy structure. As expected, the southern orientation of bamboo forests receives higher direct solar radiation in the northern hemisphere. This is consistent with studies reporting higher light availability on southern slopes due to more favorable solar incidence angles (Bátori et al., 2023; Gilliam et al., 2014; Shary et al., 2020). In contrast, the northern orientation remains predominantly shaded, where the combined shade from the canopy and bamboo clumps leads to consistently lower light intensity. The negligible fluctuations in light intensity underscore that the northern orientation exists in a stable, shaded niche sustained by diffuse radiation, with minimal direct solar radiation (Berihu et al., 2017). In the vertical dimension, incident light is progressively intercepted by the upper canopy, creating a lower-light environment for the lower leaves. Both sympodial bamboo species exhibit significant consistency in the temporal dynamics of light distribution, confirming that light heterogeneity is a common habitat characteristic rather than a species-specific effect. This stable light gradient thereby serves as a fundamental driver for the differential acclimation of leaf traits.

### 4.2 Acclimation strategies of leaf morphological traits to light heterogeneity

Interspecific differences were observed in the responses of leaf morphological traits, reflecting the resource allocation trade-offs between bamboo species. The results for LL, LW, LA, and LDW suggest that *D. farinosus* exhibits a conservative strategy in response to light heterogeneity. By maintaining stable leaf morphology, it achieves robustness in resource allocation, a strategy likely stemming from its inherent physiological regulatory capacity. Consequently, *D. farinosus* appears to acclimate to a certain degree of light variation through physiological means alone, without requiring morphological adjustments. In contrast, *B. rigida* exhibited more directed adjustments in its leaf morphology. Specifically, under the low−light conditions of the northern orientation, it increased LL, LW, and LA, thereby expanding the leaf light−capture area to compensate for the reduced photosynthetic rate caused by light insufficiency (Pires et al., 2011). Furthermore, the increased LDW observed in the upper canopy indicates greater biomass accumulation under high light, which likely contributes to a sturdier leaf structure as an acclimation to intense radiation (Guo et al., 2025). This structural reinforcement, combined with other trait adjustments, forms a coherent pattern of phenotypic plasticity that enables *B. rigida* to precisely adapt to the spatial heterogeneity of light.

Notably, both bamboo species exhibited consistent trends in LMA across orientations and canopy layers. In light-sufficient environments (e.g., southern orientation, upper canopy), elevated LMA reflects to a greater dry-mass investment per unit area. This investment is manifested in thicker, denser leaves with enhanced development of both photosynthetic and protective tissues (Ivanova et al., 2018; Leng et al., 2025), likely mitigating photoinhibition and maintaining water balance under high irradiance (Tsunoda et al., 2024; Wang et al., 2024). Conversely, under low-light conditions (e.g., northern orientation, lower canopy), the adaptive strategy shifts toward maximizing light capture and utilization (Sun et al., 2023). Here, bamboos reduce structural investment while expanding photosynthetic area to intercept more light.

### 4.3 Acclimation strategies of leaf physiological traits to light heterogeneity

In terms of chlorophyll content, the two bamboo species exhibited different acclimation patterns. In *D. farinosus*, no significant differences were found in Chl a+b content between different orientations and canopy layers. The stability of this physiological trait, in conjunction with the conservative strategy of its morphological characteristics, suggests that the species does not rely on adjusting the total amount of photosynthetic pigments to cope with fluctuations in light environment. Instead, it may maintain photosynthetic homeostasis by regulating internal light energy distribution or electron transport efficiency. The chlorophyll a/b ratio in upper canopy layers was significantly higher than that in lower layers in *D. farinosus*. This indicates that in high-light environment (upper layer), leaves of *D. farinosus* tend to invest more in chlorophyll a to enhance the conversion efficiency of reaction centers, thereby fully utilizing the abundant light energy for carbon fixation (Meneses et al, 2020). Given a stable total chlorophyll content, the lower chlorophyll a/b ratio observed in the lower layer reflects an increased proportion of chlorophyll b. This adjustment effectively enlarges the light-harvesting antenna, thereby enhancing the capacity to capture limited light energy (Herrera et al., 2022). This regulatory mechanism reflects the broad-spectrum light acclimation of *D. farinosus* by optimizing the internal configuration of the photosynthetic apparatus without altering the total pigment investment.

In contrast, *B. rigida* exhibits a typical physiological plasticity-based adaptive strategy. On northern orientation, the total chlorophyll content in leaves of *B. rigida* was significantly higher than that on the southern orientation, enhancing its efficiency in capturing and utilizing limited light by increasing chlorophyll content (Li et al., 2022). Consistent with this, the Chl a/b ratio was significantly higher on the southern orientation. This suggests that under high-light conditions, the plant minimizes light absorption by reducing the proportion of chlorophyll b and enhanced light utilization through relatively more chlorophyll a (Liu et al., 2024). Such precise adjustments in chlorophyll composition reflect the plastic adaptive strategy of *B. rigida* to achieve a dynamic balance between light capture and photoprotection under varying light conditions.

Both bamboo species exhibited enhanced photosynthetic capacity in response to greater light availability, yet they adopted divergent adaptive strategies. In both species, higher P_nmax_ was observed in high-light environment (southern orientation and upper layer). At the same time, the apparent quantum yield (AQY), a key indicator of intrinsic light-use efficiency, remained statistically stable across all positions. In *D. farinosus*, canopy layer significantly influenced LCP and R_d_, with the lowest values recorded at the north-lower position. This ability to minimize respiratory carbon loss under low-light environments, together with its maintained high P_nmax_ in high-light environments and stable AQY, supports a broad adaptive strategy. Specifically, *D. farinosus* effectively balances carbon gain in high light with carbon conservation in low light, reflecting a trade-off optimized for heterogeneous light environments (Zhang et al., 2020; Chen et al., 2011). In contrast, *B. rigida* exhibits greater plasticity in its light acclimation. Both LSP and LCP are affected by orientation, with values higher on the southern orientation than those on the northern orientation. This pattern suggests that *B. rigida* can also thrive in high-light environments by tolerating elevated respiratory costs (reflected in high LCP) in exchange for enhanced light and photosynthetic capacity (high LSP). Notably, within the southern orientation, the highest LCP and LSP occurred in the lower layer—a microhabitat characterized by sufficient but non-extreme light. This suggests that leaves in this position invest more in photosynthetic capacity while effectively avoiding photoinhibition, representing a finely tuned light acclimation strategy in *B. rigida*. Nevertheless, this high photosynthetic productivity incurs a metabolic cost, as evidenced by the elevated R_d_ in this microhabitat.

## 5 Conclusions

The spatial heterogeneity of light serves as a key environmental driver shaping the adaptive responses of both *D. farinosus* and *B. rigida*. Through an integrated analysis of morphological and physiological traits, this study reveals a clear divergence in the strategies employed by each species to cope with varying light conditions. *D. farinosus* exhibits broad environmental tolerance by maintaining stable traits, whereas *B. rigida* demonstrates precise acclimation via morphological and physiological plasticity. Both strategies represent effective acclimations to the spatial distribution of light resources, reflecting ecological mechanisms honed through long-term evolution. These findings enhance the theoretical understanding of light acclimation strategies in sympodial bamboo species.

## Data Availability

The original contributions presented in the study are included in the article/supplementary material. Further inquiries can be directed to the corresponding author.
